# Diphtheria Toxin–Producing *Corynebacterium ramonii* in Inner-City Population, Vancouver, British Columbia, Canada, 2019–2023

**DOI:** 10.3201/eid3102.241472

**Published:** 2025-02

**Authors:** Christopher F. Lowe, Gordon Ritchie, Chiara Crestani, Miguel Imperial, Nancy Matic, Michael Payne, Aleksandra Stefanovic, Diana Whellams, Sylvain Brisse, Marc G. Romney

**Affiliations:** Author affiliations: University of British Columbia, Vancouver, British Columbia, Canada (C.F. Lowe, G. Ritchie, M. Imperial, N. Matic, M. Payne, A. Stefanovic, D. Whellams, M.G. Romney); St. Paul’s Hospital, Vancouver (C.F. Lowe, G. Ritchie, N. Matic, M. Payne, A. Stefanovic, M.G. Romney); Institut Pasteur–Université Paris Cité, Paris, France (C. Crestani, S. Brisse); LifeLabs, Vancouver (M. Imperial, D. Whellams); Institut Pasteur National Reference Center for Corynebacteria of the Diphtheriae Complex, Paris (S. Brisse)

**Keywords:** *Corynebacterium ulcerans*, *Corynebacterium ramonii*, *Corynebacterium diphtheriae*, diphtheria toxin, cutaneous diphtheria, next-generation sequencing, matrix-assisted laser desorption/ionization time-of-flight mass spectrometry, MALDI-TOF MS, bacteria, zoonoses, Vancouver, British Columbia, Canada

## Abstract

We conducted patient chart reviews and whole-genome sequencing of wound specimens containing presumptive *Corynebacterium ulcerans* from Vancouver, British Columbia, Canada, during July 2019–July 2023. Sequencing confirmed 8/14 isolates were *C. ramonii* and identified 2 distinct clusters. Molecular methods should be used to clinically differentiate potential toxin-producing *Corynebacterium* spp.

*Corynebacterium ramonii*, a member of the *C. diphtheriae* species complex, has recently been differentiated from *C. ulcerans* (*1*). *C. ulcerans* is associated with zoonotic transmission (predominantly from infected cats and dogs), whereas *C. ramonii* is suspected of potential human-to-human transmission; its zoonotic character has not been established (*1*). *C. ulcerans* can manifest as respiratory or cutaneous infection similar to *C. diphtheriae* (*2*,*3*). Fourteen isolates have been previously characterized as *C. ramonii* (*1*); some harbored the diphtheria toxin gene, underscoring the clinical and public health implications of correctly identifying this organism.

In Canada, where incidence of *C. ulcerans* infections is low, a 35% increase in toxin testing referrals to the National Microbiology Laboratory in Winnipeg, Manitoba, occurred during 2006–2019; twenty-two cases of *C. ulcerans* infection were identified (45% were diphtheria toxin positive) (*4*). Although multiple reports of nontoxigenic cutaneous diphtheria (caused by *C. diphtheriae*) have occurred in the inner city of Vancouver, British Columbia, only sporadic cases of toxigenic *C. ulcerans* have been reported (*5*,*6*). Because of the additional reported *C. ulcerans* cases, most of which were subsequently identified as *C. ramonii* infections, we conducted a clinical, epidemiologic, and genomic review of *C. ramonii* infections in Vancouver. The University of British Columbia/Providence Health Care Research Institute provided ethics approval for this study (approval no. H22-03695).

## The Study

St. Paul’s Hospital (SPH) microbiology laboratory and LifeLabs in Vancouver performed a retrospective review of all specimens collected during January 2019–July 2023 that had presumptive *C. ulcerans*, according to matrix-assisted laser desorption/ionization time-of-flight (MALDI-TOF) mass spectrometry analysis. We extracted clinical data from electronic medical records for patients seen at SPH (no clinical information was available for LifeLabs’ cases), which included microbiology results, hospital course (inpatient/critical care admission), antimicrobial drugs, 30-day mortality, and risk factors for infection (housing status, substance use, and livestock/domestic animal exposure). We characterized bacterial cultures with *C. ulcerans* by using the MALDI Biotyper sirius System (Bruker, https://www.bruker.com) according to the manufacturer’s recommendations without specific extraction. We analyzed the MALDI-TOF mass spectrometry data by using the Flex Analysis function on the MALDI Biotyper (BDAL version 12, MBT Compass Library version K). At SPH, we performed antimicrobial drug susceptibility testing by using ETEST penicillin, erythromycin, clindamycin, and vancomycin gradient strips (bioMérieux, https://www.biomerieux.com) (*7*). The National Microbiology Laboratory performed all toxin testing by using PCR and modified Elek tests. For whole-genome sequencing (WGS), we extracted DNA from isolated bacterial colonies obtained from our archives by using the MagNA Pure 24 System (Roche Diagnostics, https://diagnostics.roche.com) and sequenced those on a GridION instrument (Oxford Nanopore Technologies, https://www.nanoporetech.com) by using R10.4.1 flow cells. We performed data acquisition and base calling into fast5 files by using MinKNOW version 23.07.12 and Guppy version 6.4.6 (Oxford Nanopore Technologies) and assembled raw FASTQ files by using Flye version 2.9 (*8*). We checked genome assembly quality by using QUAST v5.2.0 and analyzed assemblies by using diphtOscan (*9*,*10*). We inferred maximum-likelihood phylogeny by using IQ-TREE version 2.3.4 and a general time reversible plus gamma model; we obtained a core gene alignment by using Panaroo version 1.5.0, visualized with Microreact (https://microreact.org/project/rgLWfs1derHFm4K5ShbxgJ-c-ramonii-and-c-ulcerans-vancouver-canada) (*11*–*13*). We obtained core genome multilocus sequence typing (cgMLST) profiles by tagging the genomes for known alleles within the BIGSdb-Pasteur database (https://bigsdb.pasteur.fr/diphtheria); we used the cgMLST_ulcerans scheme. We constructed minimum-spanning trees by using GrapeTree directly from the BIGSdb-Pasteur plug-in (*14*; C. Crestani et al., unpub. data, https://doi.org/10.1101/2024.08.22.609154).

*C. ulcerans* was initially identified in cultures from 14 patients (SPH, n = 9; LifeLabs, n = 5) by using MALDI-TOF mass spectrometry (scores >2.0). Eight of those samples had a spectral peak at 5405.40 (range 5404–5407) m/z, which is associated with *C. ramonii* (*1*). All 14 isolates underwent WGS and genotyping, confirming those 8 isolates were *C. ramonii* sequence type (ST) 335 (n = 4) and ST341 (n = 4) (Figure, panel A), originating from the inner-city Vancouver (Figure, panel B). The cgMLST results suggested 2 *C. ramonii* clusters existed; isolates in cluster X had 1 allelic mismatch, and cluster Y had no allelic mismatches (Figure, panel A). We confirmed the remaining 6/14 isolates were *C. ulcerans* (ST325, ST339, ST669 [3 isolates], and ST895) (Figure, panel C). Those 6 isolates did not originate from a specific neighborhood. All 14 cultures were polymicrobial and recovered from lower extremity wounds (Table).

**Figure:**
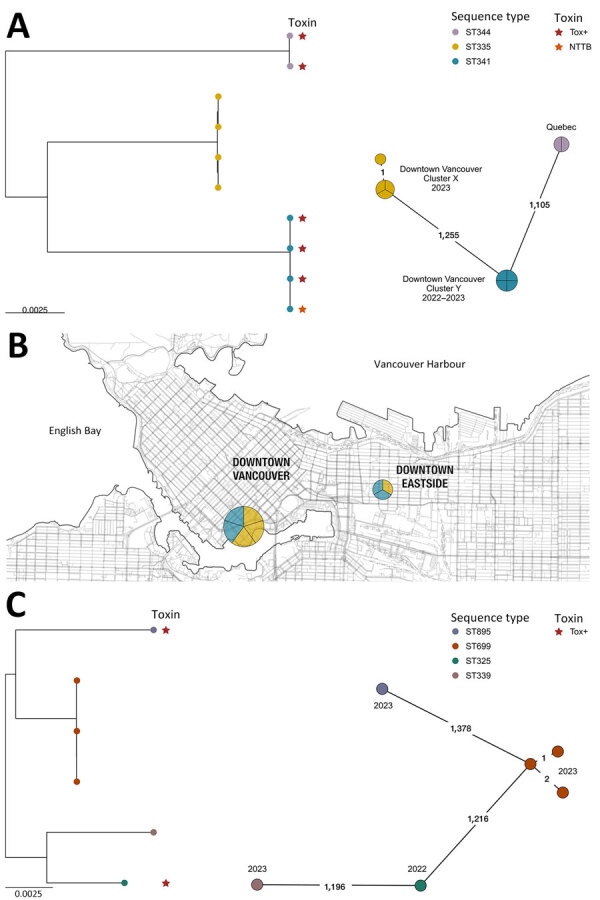
Phylogenetic analyses and location of *Corynebacterium ramonii* and *C. ulcerans* isolates in study of diphtheria toxin–producing *C. ramonii* in inner-city population, Vancouver, British Columbia, Canada, 2019–2023. Trees were rooted at midpoint. Colored circles indicate sequence type and colored stars indicate diphtheria toxin status. Numbers on lines in the spanning trees indicate number of allelic differences between sequence types; years the specimens were collected are indicated at spanning tree nodes. A) Maximum-likelihood phylogeny (left side) and minimum spanning tree (right side) of 8 *C. ramonii* isolates from this study (British Columbia) together with 2 isolates, LSPQ-04227 and LSPQ-04228, from Quebec, Canada (*1*); 2 clusters of infections can be observed, corresponding to ST335 and ST341 isolates. B) Spatial map of *C. ramonii* isolates from this study, all originating from downtown Vancouver. C) Maximum-likelihood phylogeny (left side) and minimum spanning tree (right side) of 6 *C. ulcerans* isolates from this study (British Columbia). Scale bars indicates nucleotide substitutions per site. NTTB, nontoxigenic *tox* gene–bearing; ST, sequence type; Tox+, *tox* gene and toxin both present.

**Table:** Antimicrobial drug susceptibility, co-isolated bacteria, and toxin test results for wound specimens containing *Corynebacterium ulcerans* and *C. ramonii* collected in Vancouver, British Columbia, Canada, 2019–2023*

Characteristics	*Corynebacterium ulcerans*, n = 6	*Corynebacterium ramonii*, n = 8
Antimicrobial drug susceptibility		
Penicillin	NA	Intermediate, n = 8
Erythromycin	NA	Susceptible, n = 8
Clindamycin	NA	Susceptible, n = 1; intermediate, n = 7
Vancomycin	NA	Susceptible, n = 8
Frequency of co-isolated bacteria from wound cultures	Enterobacterales, n = 4; MSSA, n = 3; coagulase-negative staphylococci,† n = 2; β-hemolytic *Streptococcus,*‡ n = 2*; Acinetobacter baumannii,* n = 1	β-hemolytic *Streptococcus,*§ n = 6; *Arcanobacterium hemolyticum,* n = 4; MSSA, n = 4; Enterobacterales, n = 3; yeast, n = 1*; Pasteurella multocida,* n = 1
Toxin testing, no. (%) isolates		
Nontoxigenic	4 (66.6)	4 (50.0)
Nontoxigenic, *tox* gene–bearing	0	1 (12.5)
Toxigenic	2 (33.3)	3 (37.5)

*Bacteria were isolated from wounds on lower extremities of patients. MSSA, methicillin-susceptible *Staphylococcus aureus*; NA, not applicable. †*Staphylococcus pseudintermedius, S. lugdunensis*. ‡*Streptococcus dysgalactiae* and group G *Streptococcus*. §*Streptococcus pyogenes,* *S. dysgalactiae* subspecies *equisimilis*.

Three of 8 patients with *C. ramonii* infection required admission to the hospital (average duration 6 days); critical care admissions were not required, and we observed no 30-day mortality. Toxigenic systemic signs were not observed in the 3 patients infected with diphtheria toxin-producing *C. ramonii*. Of the 6 patients who had electronic medical records, all were treated with antimicrobial drugs (piperacillin/tazobactam, amoxicillin/clavulanate, trimethoprim/sulfamethoxazole, cephalexin, cefazolin, or ceftriaxone). All 8 patients with *C. ramonii* infections were associated geographically within downtown Vancouver (Figure, panel B), including 5 persons experiencing homelessness. However, *C. ulcerans* cases were distributed throughout the city outside of downtown (according to postal codes). All patients with *C. ramonii* infections reported a history of substance use disorder, and none had documented livestock or domestic animal exposure.

## Conclusions

*C. ramonii* cases clustered exclusively within downtown inner-city Vancouver, whereas *C. ulcerans* cases occurred outside of the city’s downtown core. Human-to-human transmission of *C. ramonii* has been hypothesized (*1*), and our findings provide evidence for possible human-to-human transmission. WGS showed 2 distinct *C. ramonii* clusters (ST335 and ST341) within the same community. Vancouver’s downtown core has high rates of poverty, persons experiencing homelessness, and substance abuse, and persons might transmit bacteria via close contact, such as that observed for a previous cutaneous *C. diphtheriae* cluster (*5*). *C. ulcerans* infections are associated with zoonotic transmission; however, animal exposures were not observed for patients with *C. ramonii* infections. *C. ramonii* was identified in wounds along with other bacteria associated with human reservoirs, such as *Streptococcus pyogenes*, *S*. *dysgalactiae* subspecies *equisimilis*, and *Arcanobacterium hemolyticum*. The clinical manifestations of *C. ramonii* infections align more closely to those of cutaneous *C. diphtheriae* than to those of *C. ulcerans* infections (*5*).

Our study highlights the role for MALDI-TOF mass spectrometry identification of *C. ramonii*; the initial description of *C. ramonii* reported a unique peak at 5405.40 m/z (*1*). In this study, *C. ramonii* identification required WGS; however, a retrospective review of spectra confirmed the presence of the 5405.40 m/z peak, although the range was broader. MALDI-TOF mass spectrometry is used widely in clinical laboratories, and *C. ramonii* prevalence can be more accurately estimated when peak analysis of mass spectrograms is performed to avoid misidentification as *C. ulcerans*. Continual updating of MALDI-TOF mass spectrometry databases is also needed to enable accurate detection of emerging pathogens, such as *C. ramonii*. Although 16S rRNA gene sequence analysis has been increasingly used in clinical laboratories, it has not been as effective as *rpoB* gene sequence determinations for differentiating between *Corynebacterium* spp. (*1*).

The first limitation of our study is the relatively small number of cases available for analysis. Because wound cultures were polymicrobial, it is unclear to what degree *C. ramonii* contributed to wound infections. Second, we could not perform a formal case–control study; patients with *C. ulcerans* infection were primarily seen in outpatient clinics outside of our healthcare network, and complete medical records (including animal contact histories) were not accessible.

In conclusion, our findings correlate with the initial clinical description of *C. ramonii*, which appears to manifest symptoms similar to those of cutaneous *C. diphtheriae* infections. Using molecular methods, such as WGS or manual MALDI-TOF mass spectral analysis, will be needed to clinically differentiate between *C. ulcerans*, *C. ramonii*, and other members of the *C. diphtheriae* species complex.
